# Antioxidant, Anti-Tumour, and Anticoagulant Activities of Polysaccharide from *Calocybe indica* (APK2)

**DOI:** 10.3390/antiox11091694

**Published:** 2022-08-29

**Authors:** Ambika Nataraj, Sudha Govindan, Prasanna Ramani, Krishnamoorthy Akkana Subbaiah, S. Sathianarayanan, Baskar Venkidasamy, Muthu Thiruvengadam, Maksim Rebezov, Mohammad Ali Shariati, José M. Lorenzo, Mirian Pateiro

**Affiliations:** 1 Department of Biochemistry, School of Biosciences, Periyar University, Salem 636011, India; 2 Dhanvanthri Laboratory, Department of Sciences, Amrita School of Physical Sciences, Amrita Vishwa Vidyapeetham, Coimbatore 641112, India; 3 Center of Excellence in Advanced Materials & Green Technologies (CoE–AMGT), Amrita School of Engineering, Amrita Vishwa Vidyapeetham, Coimbatore 641112, India; 4 Department of Plant Pathology, Tamil Nadu Agricultural University, Coimbatore 641003, India; 5 Faculty of Pharmacy, Karpagam Academy of Higher Education, Coimbatore 641021, India; 6 Department of Oral & Maxillofacial Surgery, Saveetha Dental College and Hospitals, Saveetha Institute of Medical and Technical Sciences (SIMATS), Saveetha University, Chennai 600077, India; 7 Department of Crop Science, College of Sanghuh Life Science, Konkuk University, Seoul 05029, Korea; 8 Department of Scientific Research, V. M. Gorbatov Federal Research Center for Food Systems, 26 Talalikhin st., 109316 Moscow, Russia; 9 Biophotonics center, Prokhorov General Physics Institute of the Russian Academy of Science, 38 Vavilov st., 119991 Moscow, Russia; 10 Semey Branch of the Institute, Kazakh Research Institute of Processing and Food Industry, 238«G» Gagarin Ave., Almaty 050060, Kazakhstan; 11 Centro Tecnológico de la Carne de Galicia, Avd. Galicia No. 4, Parque Tecnológico de Galicia, San Cibrao das Viñas, 32900 Ourense, Spain; 12 Área de Tecnoloxía dos Alimentos, Facultade de Ciencias, Universidade de Vigo, 32004 Ourense, Spain

**Keywords:** *Calocybe indica*, polysaccharides, antioxidant, anticoagulant, antiproliferative activity, edible mushroom

## Abstract

The initial structural features and in vitro biological study of crude polysaccharides from *Calocybe indica* (CICP) extracted by hot water followed by ethanol precipitation was investigated. High-performance gel permeation chromatography, HPLC-DAD, UV, IR and NMR spectroscopy, X-ray diffraction, scanning electron microscopy, and Congo red methods were used to determine structural features. The results revealed that CICP is a hetero-polysaccharide with a molecular weight of 9.371 × 10^4^ Da and 2.457 × 10^3^ Da which is composed of xylose, mannose, fucose, rhamnose, arabinose, galactose, and glucose. The antioxidant activity of CICP was evaluated using radical scavenging activity (three methods), reducing ability (three methods), metal chelating activity, and lipid peroxidation inhibition activity (two methods). It was found that the antioxidant capacity is concentration-dependent and EC_50_ values were found to be 1.99–3.82 mg/mL (radical scavenging activities), 0.78–2.78 mg/mL (reducing ability), 4.11 mg/mL (metal chelating activity), and 0.56–4.18 mg/mL (lipid peroxidation inhibition activity). In vitro anticoagulant assay revealed that CICP could prolong activated partial thromboplastin time (APTT), thrombin time (TT), but not prothrombin time (PT). CICP exhibited antiproliferative activity on HeLa, PC3, HT29, HepG2, and Jurkat cell lines with IC_50_ (μg/mL) values of 148.40, 143.60,151.00, 168.30, and 156.30, respectively. The above findings suggested that CICP could be considered a natural antioxidant and cancer preventative.

## 1. Introduction

Every organism generates reactive oxygen species (ROS) such as hydroxyl radical, superoxide radical, or hydrogen peroxide [1] throughout cellular oxygen metabolism. While normal cellular physiology demands a balance of ROS production and clearance, pathogenic conditions cause an imbalance. The overabundance of reactive oxygen species (ROS) can induce oxidative stress in DNA, proteins, and lipids, leading to disorders such as diabetes, atherosclerosis, cancer, and cardiovascular disease [2]. To neutralise ROS, all organisms have a sophisticated antioxidant defence system that includes both enzymic and non-enzymic antioxidant systems that create antioxidant enzymes and metabolites. On the other hand, natural defence systems are inadequate to totally stop oxidative damage. More study is being conducted to develop effective, non–toxic exogenous antioxidants that can help avoid oxidative injury and contribute to redox equilibrium [3].

Mushrooms are valued for their nutritive and non-poisonous therapeutic characteristics, as well as food and medicinal resources. Many antioxidant substances, including polysaccharides, phenolics, ergothioneine, tocopherols, and carotenoids, have been identified in their fruiting bodies, mycelium, and fermentation broth [4]. Over the last decade, mushroom polysaccharide research has progressed in various ways, and it has been suggested as a promising contender in the hunt for natural antioxidants that are both effective and non-toxic. Edible mushrooms have a wide range of medicinal and bioactivities. Polysaccharides, highly available biopolymers, are essential active ingredients in consumable mushrooms, and numerous research has shown that polysaccharides obtained from a variety of mushrooms have extensive bioactivities. *Oudemansiella radicata* [5], *Antrodia cinnamomea* [6], *Paxillus involutus* [7], and *Armillaria osyoyae* [8] have been demonstrated to have powerful antioxidant effects on oxygen radicals. Various polysaccharides from *Pleurotus eryngii* [9], bachu mushroom [10], *Tricholoma mongolicumImai* [11], *Lentinus crinitus* [12], and *Marasmiellus palmivorus* [13] have shown antiproliferative activities. Polysaccharides from *Pleurotus eous* [14], *Agrocybe aegerita* [15], and *Catahelasma ventricosum* [16] exhibited anticoagulant activities. The extraction method could have an important impact on polysaccharide yield and structural characteristics, as well as biological activities. Because of its ease of use and environmental friendliness, hot water extraction, a traditional technology, has been generally utilised for the extraction and processing of polysaccharides since ancient times. It is the most convenient and traditional approach widely used in industry [17].

*Calocybe indica* (milky mushroom) is an edible mushroom native to India that belongs to the order Agaricales and the Lycophyllaceae family. Secondary metabolites such as phenolic chemicals, terpenes, and steroids are found in it, and they may have a role in its therapeutic and nutritional characteristics [18]. *C. indica* var. APK2 yielded a water-soluble polysaccharide with immuno-stimulatory and cytotoxic properties [19]. In d-galactose-induced aging mice, *C. indica* polysaccharide had substantial antioxidant effects and an anti-aging impact by restoring antioxidant enzyme activity and reducing lipid peroxidation [20]. Methanolic extracts of *C. indica* have been demonstrated to have in vitro antioxidant activity in several chemical assays, with the results revealing that the stipe of *C. indica* is more promising than the cap [21].

To our knowledge, no comprehensive study of the physicochemical characteristics and biological investigation of polysaccharides from *C. indica* has been conducted, nor has the relationship between chemical structure and antioxidant activity of CICP been investigated. As a result, the goal of this work was to extract polysaccharides from *C. indica* utilising the hot water extraction method. We evaluated the chemical profile, monosaccharide composition, molecular weight, surface morphology, and other preliminary structural aspects. The antioxidant, anticoagulant, and anti-tumour effects of polysaccharides were also studied in vitro, as well as the structure–activity relationship.

## 2. Materials and Methods

### 2.1. Reagents

*Calocybe indica* fruiting bodies were freshly picked from a mushroom plantation in Coimbatore, Tamil Nadu, India. Human cervical cancer (HeLa), liver cancer (HepG2), colon adenocarcinoma (HT29), prostate cells (PC3), and T-lymphocyte (Jurkat) cells were obtained from the National Centre for Cell Science of India. Detection kits for anticoagulation studies were procured from Agappe diagnostic Ltd. Gibco, Invitrogen provided Dulbecco’s modified Eagle’s medium (DMEM), fetal bovine serum (FBS), penicillin, and streptomycin. For use in the present study, 1,1-diphenyl-2-picrylhydrazyl, *N, N*-dimethyl-o-phenylenediamine, neocuproine, phenanthroline, β-carotene, linoleic acid, 3-(4,5-dimethylthiazol-2-yl)- 2,5-diphenyltetrazolium bromide, monosaccharide standards, and molecular mass standards were acquired from Sigma-Aldrich (India). The rest of the chemicals, such as solvents, acids, bases, and reagents, were of analytical quality.

### 2.2. Hot Water Extraction

The delipidated *C. indica* fruit body powder was processed thrice at 100 °C for three hours using thirty volumes of distilled water [22]. Combined, the extracts were filtered, evaporated under vacuum, centrifuged (4500× *g*) for 15 min, and precipitated (ethanol (95%, *v*/*v*) at 5 °C) to extract polysaccharides. After extracting the proteins with Sevag’s reagent (chloroform: butanol = 4:1), the water phase was dialyzed with de-ionized water (three days) to eliminate salts and other contaminations. To obtain the *C. indica* crude polysaccharides, the dialysate was condensed by rotary evaporation and then freeze-dried (CICP).

### 2.3. Structural Characterization

#### 2.3.1. Total Carbohydrate, Protein, Uronic Acid, and Sulfate

According to [23], the neutral sugar content of CICP polysaccharides was determined using the phenol–sulfuric acid technique. An amount of 1 mL 5% phenol solution was added to each 2 mL of CICP. After that, 5 mL sulfuric acid was added and thoroughly mixed. We left the mixture at 30 °C for 20 min. The absorbance was detected at 490 nm. By using glucose as a standard solution, a calibration curve was created.

The Bradford method [24] was used to determine the protein content. In a test tube, 1 mL of CICP was added, followed by a 5 mL solution of Coomassie bright blue G250. The optical absorbance was measured at 595 nm after incubation for 5 min. Bovine serum albumin was used to perform the calibration.

The uronic acid content was measured using the carbazole method [25]. An amount of 1.0 mL CICP was mixed with 5.0 mL sodium tetraborate sulphuric acid solution (9.54 mg/mL) and heated for 10 min in a boiling water bath. The mixture was cooled and then we added 0.2 mL of carbazole ethanol solution (1.25 mg/mL) and heated for another 10 min. The absorbance was measured at 530 nm in comparison to a reagent blank. The calibration curve was made with galacturonic acid.

Sulfate content was measured by the barium chloride/gelation method after acid hydrolysis (1 M HCl for 6 h at 100 °C) [26]. To 0.2 mL of CICP, we added 3.8 mL of 4% TCA, followed by 1.0 mL of the barium chloride gelatin reagent. It was mixed and allowed to stand for 10–20 min at room temperature at 360 nm against a reagent blank. The calibration curve was constructed using potassium sulphate.

#### 2.3.2. UV, FT-IR, and NMR

UV spectra of CICP (2 mg/mL) in the range of 200–800 nm were recorded on a UV spectrophotometer (UV-1800, Shimadzu, Kyoto, Japan). Thermo-Scientific Nicolet (5700IR) was used to take IR spectrum. A 600 MHz spectrometer (Bruker AVII 500 MHz) was used to record nuclear magnetic resonance (NMR) spectroscopy in D_2_O.

#### 2.3.3. Congo Red Binding Assay

Nearly 2 mL of polysaccharide (6 mg/2 mL) was combined with 2.0 mL Congo red (0.1 mmol/L) in NaOH (0–0.5 mol/L) at different concentrations [27]. Incubated for 30 min, and spectrophotometer (Shimadzu UV-1800) was used to determine the absorbance at 400 to 700 nm and record the maximum absorbance. Distilled water was used as a control.

#### 2.3.4. Molecular Weight

The molecular mass of CICP was found by high-performance size-exclusion chromatography fitted with Shodex SB-804 HQ column (300 × 8.0 mm, Showa Denko Corp., Tokyo, Japan) and refractive index detector. During each run, a 10 μL sample (2 mg/mL) was injected with a flow speed of 0.9 mL/min and the column at 45 °C. The molecular weight of CICP was examined by associating the retention time of a standard curve constructed using a series of dextran standards.

#### 2.3.5. Monosaccharide Composition

Using 1-phenyl-3-methyl-5-pyrazolone (PMP) derivation and HPLC (Agilent, Santa Clara, CA, USA) analysis, the monosaccharide content of CICP was determined [28]. CICP (10 mg) was hydrolysed with 4 M trifluoroacetic acid (TFA) for four hours. After the hydrolysis process was completed, the excess of TFA was eliminated using three rounds of co-distillation with methanol and steam of nitrogen. The dried sample dissolved in ultrapure water was centrifuged for 10 min, and the supernatant was used for further derivatisation. Approximately 25 µL of hydrolysed CICP solution was derivatised for 100 min in a water bath at 70 °C with 50 µL of 0.6 M NaOH and 50 µL of methanolic PMP solution (0.5 M). The mixtures were added to 50 µL of 0.3 M HCl solution after cooling at RT in order to terminate the reaction. This was followed by extraction with 1 mL each of water and chloroform three times. After discarding the chloroform layer, the extraction procedure was carried out three more times. For HPLC analysis, the aqueous layer was filtered using a 0.22 µm membrane filter. The sample was eluted using a Zorbax Eclipse Plus C18 column (250 mm × 4.6 mm × 5 µm), a 5 µL injection volume, a temperature of 30 °C, and a mixture of 17 = % acetonitrile and 83% of 0.025 M potassium phosphate buffer (pH, 6.7) at a flow rate of 0.7 mL/min. The UV absorbance was examined by a diode array detector (DAD; Agilent, Santa Clara, CA, USA) at 250 nm. The preparation and process were the same for the monosaccharides (Gal, Glc, Rha, Rib, Fuc, Man, Ara, Xyl, GalA, and GlcA).

#### 2.3.6. X-ray Diffraction Analysis and Scanning Electron Microscopy (SEM)

To detect any changes in the crystal structure of CICP, an X-ray diffractometer was used with a Cu-K monochromatic radiation source and a Ni filter with a scan speed of 10°/min and the diffraction angle changed from 2 = 5° to 2 = 70°. For SEM, the dried sample was sputtered with gold (100 nm thickness) on an SEM stub before being examined with a scanning electron microscope.

### 2.4. In Vitro Antioxidant Activity

#### 2.4.1. Free Radical Scavenging Activity

##### ABTS Cation Radical Scavenging Behaviour

The ABTS+ cation radical scavenging activity of CICP was evaluated using the method described previously [29]. In brief, the ABTS+ cation was created by combining 2.45 mM potassium persulfate with 7 mM ABTS cation solution and incubating for 16 h in the dark at room temperature (25 °C). The ABTS+ cation solution was diluted to an absorbance of 0.700 ± 0.02 at 734 nm using 80% ethanol. An amount of 10 µL of 1–5 mg/mL of CICP was combined with 1 mL of diluted ABTS+ cation solution, and after 6 min of mixing, the absorbance was measured at 734 nm. The quenching activity of ABTS+ cation radical is computed as follows: (A0 − A1)/A0] × 100, where A0 is the absorbance of ABTS + water and A1 is ABTS^+^ CICP.

##### Analysis of Superoxide Radical Scavenging Activity

The superoxide radical trapping activity was executed by pyrogallol autoxidation method [30]. In short, approximately 4.5 mL of Tris-HCl buffer (0.05 mol/L, pH8.2), 0.5 mL pyrogallol solution (2.5 mmol/L, pH6.0), and 1 mL CICP (1–5 mg/mL) were mixed, incubated (25 °C, 5 min), then stopped with hydrochloric acid solution (1 mL, 8.0 mol/L). The absorbance was monitored at 320 nm. Vc was selected as the positive control. The scavenging capacity is evaluated as (A0-A1/A0) x 100, where A0 = control absorbance and A1 = sample absorbance.

##### DMPD Radical Scavenging Activity

The decrease in the purple-coloured radical DMPD+ is the basis for this test and is performed using [31] methodology. An amount of 20.9 mg of DMPD was solvated in 1 mL de-ionized H_2_O to make DMPD^+^ (100 mM), approximately 500 μL of the above mixture was supplemented to 50 mL of acetate buffer (0.1 M, pH 5.25), and the coloured radical cation generated by the addition of 0.1 mL of a 0.05 M ferric chloride solution, and the reagent was adjusted to an absorbance of 0.900 at 505 nm. The reagent was prepared fresh and was stable for 12 h. The test tubes were filled with various amounts of CICP (0.5–2.5 mg/mL) and reference antioxidant (Trolox), and the total volume was made to 0.5 mL using distilled water. After 30 min of incubation, DMPD^+^ (1 mL) was introduced to the solution, and absorbance was recorded at 505 nm. As a blank, a buffer solution was used. The results were determined using the same formula that was utilized in the earlier process.

#### 2.4.2. Antioxidant Activity Based on Metal Chelation

Dinis’ method [32] was used to test ion chelating activity using the ferrozine assay. Ferrous chloride (0.1 mL, 2 mM) and 3.7 mL of water were combined with CICP (1–5 mg/mL), followed by adding ferrozine (0.2 mL, 5 mM) and letting it sit for 10 min (RT). The reaction mixture’s absorbance was estimated at 562 nm. The ratio of inhibition of the development of the ferrozine–Fe^2+^complex was determined as follows: A_control_ − A_sample_/A_control_ × 100; where A_control_ = absorbance ofFeCl_2_ ferrozine; A_control_ = absorbance of CICP. In this assay, the reference was ethylene diamine tetraacetic acid (EDTA).

#### 2.4.3. Reducing Ability of Antioxidant Assays

##### Cu(II) Reduction Capacity Assay

The CUPRAC assay measured the sample’s capacity to reduce Cu^2+^ to Cu^+^ using a chelating agent such as neocuproine [33]. In a test tube, CuCl_2_ (0.25 mL, 0.01 M), ethanolic solution of neocuproine (0.25 mL, 7.5 × 10^−3^ M) and ammonium acetate buffer (0.25 mL, 1 M) were mixed with varying concentrations of CICP (0.5–2.5 mg/mL). We made up the total volume to 2 mL with distilled water, incubated (30 min, RT) and measured the absorbance (450 nm) against a reagent blank.

##### Ferric Reducing Antioxidant Power Assay (FRAP)

We prepared FRAP working reagent in the following sequence order: Acetate buffer (25 mL, 0.3 mol/L, pH 3.6), TPTZ (2.5 mL, 10 mmol/L in 40 mM HCl), and FeCl_3_·6H_2_O (2.5 mL, 20 mmol/L). We mixed 900 μL FRAP reagent, 90 μL water, and 30 μL CICP (0.5–2.5 mg/mL) or Trolox and incubated (30 min, 37 °C). Escalated reaction absorbance (595 nm) meant augmented reducing ability compared to that of Trolox reference [34].

##### Phenanthroline Assay

This test quantifies the change in absorbance based on the formation of the ferrous phenanthroline complex. Amounts of 0.10 mL CICP or BHT, ferric chloride (0.50 mL, 0.2%), and 1, 10-phenanthroline (0.25 mL, 0.5%) were combined and the total volume made up to 5 mL with methanol. After 20 min of incubation at 30 °C in darkness, the absorption (510 nm) of the orange-red colour was estimated [35].

#### 2.4.4. Anti-Lipid Peroxidation Activity

##### Lipid Peroxidation Inhibition Assay

The lipid peroxide produced was measured by means of an altered thiobarbituric acid reactive species (TBARS) assay with egg yolk homogenates as lipid-rich media [36]. In a test tube, 10% egg homogenate (0.5 mL) and 0.1 mL of CICP (1–5 mg/mL) were combined, and the capacity was increased to 1.0 mL by adding water. In order to cause lipid peroxidation, FeSO_4_ (0.05 L, 0.07 M) was applied to the above solution and incubated (30 min). Subsequently, 20% acetic acid (1.5 mL, pH modified to 3.5 with NaOH), 0.8% TBA (1.5 mL in 1.1% sodium dodecyl sulphate), and 20% TCA (0.05 mL) were introduced, vortexed and warmed in a water bath (1 h) [37]. After cooling, each volume was made up with n-butanol (5 mL) and centrifuged (10 min, 3000 rpm). The organic upper layer’s absorbance was estimated at 532 nm. As a positive control, Trolox was used.

##### β-Carotene Bleaching Assay (BCBA)

The linoleic acid/β-carotene combination was utilized to assess the anti-lipid peroxidation activities of the samples [38]. We prepared a β-carotene solution (2 mg/10 mL chloroform) and transferred 2 mL into a flask and swirled to mix with linoleic acid (40 mg) and Tween 40 (400 mg). After removing CHCl_3_, added water (100 mL), and the suspension was agitated strenuously. We pipetted out 2.4 mL of the blend into a diverse test tube comprising 0.1 mL of CICP (1–5 mg/mL). Trolox was used as the positive control. CICP was substituted with water in the control group. The zero-time absorbance was noted at 470 nm by a spectrophotometer as soon as the sample was added to each tube. The tubes were then incubated at 50 °C in a hot water bath. After 2 h, the absorbance was measured again. As a positive control, BHA was used. For background subtraction, a blank free of β-carotene was developed. Bleaching inhibition (%) = (β-carotene content after 2 h of assay/initial β--carotene content) × 100.

Radical scavenging activities such as ABTS [23], superoxide [14], DMPD [21] were performed as per the procedure reported. Metal chelation antioxidant assay was performed using the reported protocol [24]. Reducing ability antioxidant assays such as copper reduction capacity assay [14], ferric reducing antioxidant power assay (FRAP) [25], and phenanthroline assay [26] were performed in accordance with the procedure reported. Anti-lipid peroxidation activities, such as lipid peroxidation inhibition assay [27] and β-carotene bleaching assay (BCBA) [28], were performed as per the procedure reported in the literature.

### 2.5. Anticoagulation Activity

As previously mentioned, ref. [39] APTT, PT, and TT were determined using an automatic coagulation analyser (Herba Transasia Biomedical Ltd.) with APTT, PT, and TT reagents and standard human plasma.

### 2.6. Anti-Proliferation Assay

CICP’s antiproliferative effects on various cell lines were assessed using the MTT test [40] with cisplatin as a standard. Cells were implanted in 96-well microplates (5 × 10^3^ cells/mL) in DMEM media with 10% FBS, 100 U/mL penicillin, and streptomycin. The cells were combined with numerous CICP dilutions and treated at 38 °C (1 day). Successively, MTT reagent (20 μL, 5 mg/mL) was appended to each well. Incubated (4 h), separated, and DMSO was used to dissolve formazan. An ELISA reader was used to measure the absorbance (570 nm).

### 2.7. Statistical Analysis

All the studies were performed in triplicate, and the outcomes are shown as means with standard deviations (SD). One-way analysis of variance (ANOVA) was utilized using SPSS software. Duncan’s multiple range test was used to calculate the differences in the means. All values with a *p* < 0.05 were considered statistically significant.

## 3. Results and Discussion

### 3.1. Physico-Chemical Characteristics of CICP

The chemical composition (CICP) was estimated, as shown in Table 1. Hot water extraction, alcohol precipitation, deproteination, and dialysis were used to separate crude polysaccharides obtained from *C. indica* to minimise the damage that could affect their structure. Table 1 includes the physicochemical properties of the crude polysaccharides. Carbohydrates made up the majority of CICP (79.29%), with a small amount of protein (2.15%), uronic acid (4.40%), and sulfate (4.70%). The carbohydrate content was comparable to that of *Flammulina velutipes* polysaccharide (FVPU) (80.65%) [41] and *Lentinus edodes* polysaccharide (78.20%) [42], higher than that of crude *Lepista nuda* polysaccharides (70.60%) [43], *Coriolus versicolor* polysaccharides (72.40%) [44], and *Ganoderma applanatum* polysaccharides (63.50%) [42], and lower than that of *Trametes versicolor* and *Flammulina velutipes* polysaccharides (FVPH) (83.90% and 84.86%, respectively) [41,42]. The protein content was comparable to that of crude *Ganoderma lucidum* polysaccharide (2.70%) and lower than that of *Paxillus involutus* polysaccharide (PIP2-1) [7]. Shu et al. [43] reported a sulphate content of 3.39%, which is lower than that of CICP. The uronic acid concentration of CICP was lower than that of *Monascus purpureus mycelium* crude polysaccharide (MPS) (7.57%) and higher than water (MPS-1) (0.98%) and 0.2 M NaCl eluted fractions (MPS-2) (1.53%) [45]. The uronic acid content of polysaccharide has been found to be closely connected to their antioxidant capabilities [46]. As a result of the obvious carbohydrate and uronic acid concentration, the antioxidant capacity may be increased.

### 3.2. Structural Characterization of CCIP

**Figure 1:**
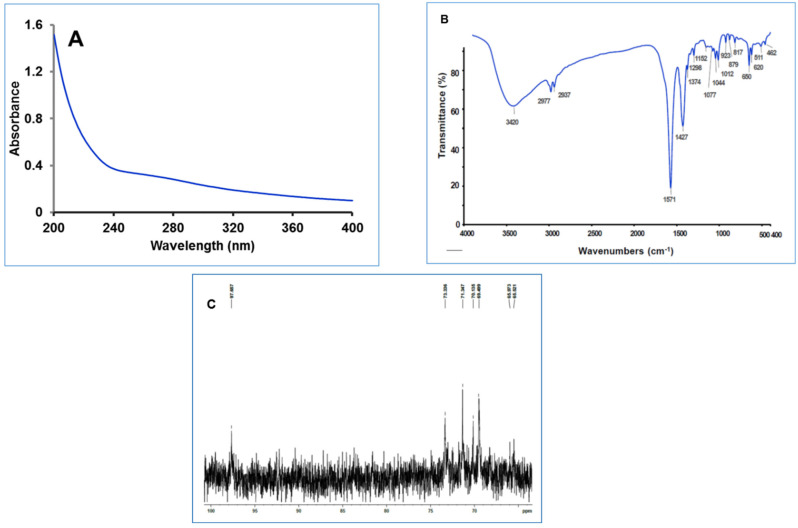
UV spectra of CICP (**A**); FTIR spectra of CICP (**B**); ^13^C spectra of CICP (**C**).

On the UV spectrum, there was minute absorption at 280 nm, confirming little protein in the polysaccharide (Figure 1A).

In the case of polysaccharides, FT-IR spectroscopy is commonly used to detect various functional groups (C-H, N-H, O-H, and C-O, Figure 1B). The hydroxyl O-H stretching vibration was represented by the distinctive broad peak at 3420 cm^−1^. The weak band at 2977 cm^−1^ is linked to hydroxyl group stretching vibrations, as well as C-H stretching and bending vibrations [47]. The presence of amide group vibrations at 1537 cm^−1^ indicates that these polysaccharides contain bound protein. Sulfate groups (SO_3_^−^) absorption peaks were also found at 1252 cm^−1^ in support of an asymmetrical S=O stretching vibration [48]. CICP’s signal at 1374 cm^−1^ revealed the distinctive absorption of C-H bonds, whereas CICP absorption peak at 1427 cm^−1^ indicated the stretching vibration of C-O and C-H [49]. The absorption peaks in the 1200–1000 cm^−1^ range are created by C–O stretching vibrations, 1152, 1077, 1044, and 1012 cm^−1^ being ring vibrations overlapping C–O–H side branch stretching vibrations and C–O–C glycosidic bond vibrations, respectively [26], suggesting that CICP contained pyranose monomers. Furthermore, the absorption at 923 and 879 cm^−1^ suggested the presence of α-glycosidic and β-glycosidic bonds, respectively [50]. The signal at 860 cm^−1^ corresponds to β-glycoside linkages, which are the polysaccharide’s major active components and indicate bioactivity. As a result, it was deduced that CICP had both α-and β-configurations at the same time.

Generally, the ^1^H NMR of polysaccharides will be packed in the aliphatic region due to the presence of various sugar units (Appendix A). Likewise, we found that signals for CCIP are crowded in the regions between δ 3.367 and 5.295 ppm. Irrespective of all the signals, the anomeric hydrogens (both α and β anomers) of CCIP are found at 4.971 and 5.117 ppm, and their corresponding carbon signals are observed in carbon NMR. The methyl hydrogens are observed at 1.141 and 1.233 ppm, which corresponds to 65.52 and 65.97 ppm in carbon NMR (Figure 1C).

### 3.3. Molecular Weight and Monosaccharide Composition

**Figure 2:**
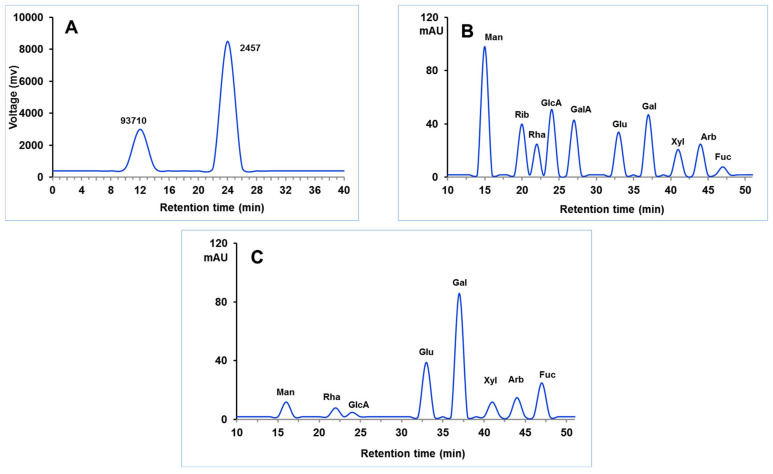
HPSEC chromatograph of CICP (**A**); HPLC chromatogram of standard monosaccharide (**B**) and CICP (**C**).

On the HPSEC chromatograph, CICP revealed two asymmetric peaks, indicating that it is heterogeneous (Figure 2A). The average M.wt of CICP was, thus, calculated to be 9.371 × 10^4^ Da and 2.457 × 10^3^ Da based on the standardization curve executed by several dextran M.wt standards. *Ramaria botrytis* (Pers.) purified fraction RBP-1 and RBP-3 have molecular weights of 6.48 and 96.72 kDa, respectively, which is consistent with our findings. The M.wt range for crude polysaccharides from thirteen Boletus mushrooms ranged from 2.66 × 10^3^ Da to 1.94 × 10^7^ Da. The M.wt of CICP was lower than that of *Ganoderma lucocontextum* (GLP-3) and *Auricularia auricula* (AAP–3–1) [51], which were both reported to be 159.7 kDa and 320.9 kDa, respectively. CICP was found to be constituted of xylose, arabinose, fucose, rhamnose, mannose, galactose, glucose, and glucuronic acid with concentrations of 3.06%, 3.58%, 5.55%, 1.15%, 2.93%, 66.23%, 15.52%, and 1.98%, respectively, according to HPLC analysis (Figure 2B). The two most prevalent monosaccharides in the structure of CICP’s backbone were observed to be high in galactose and glucose, respectively. The findings strongly showed that CICP is a high-molecular-weight hetero galactan. On conventional hot water extracted polysaccharides, sugar composition analysis revealed that galactose is the most prevalent sugar, followed by glucose and fucose, supporting the current study [52]. According to the current study, the neutral polysaccharides derived from white *Hypsizygus marmoreus*, *Flammulina velutipes*, *Pleurotus ostreatus,* and *Pleurotus eryngii* had galactose (42.7~69.1%) as the predominant monosaccharide [53].

### 3.4. X-ray Diffraction

**Figure 3:**
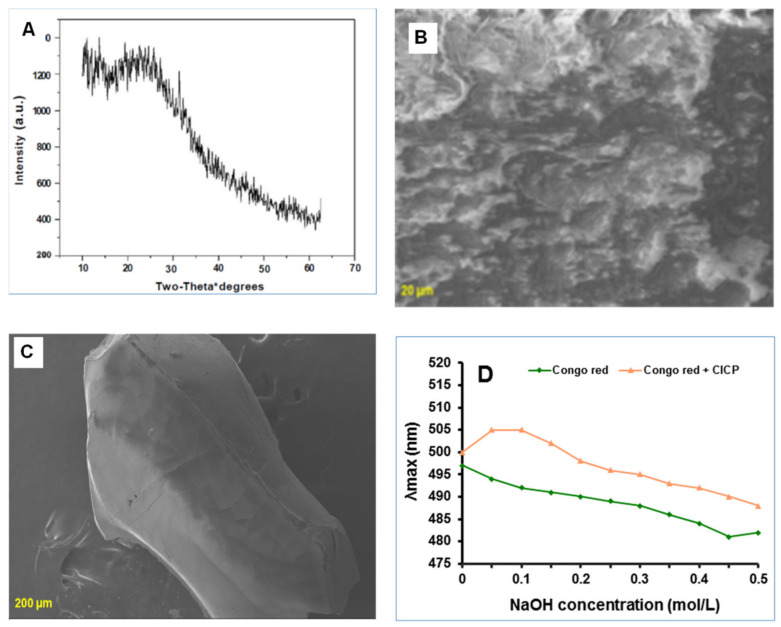
XRD pattern of CICP (**A**); SEM image of CICP (**B**,**C**); change in λmax of CICP at various NaOH concentrations (**D**).

As demonstrated in Figure 3A, the XRD pattern of CICP was between 10° and 62°. The curve is broad and lacks sharp peaks, indicating CICP’s amorphous nature, which is consistent with earlier research [54].

### 3.5. Scanning Electron Microscopy

The utmost well-organized tool for analysing the surface structure of biopolymers is scanning electron microscopy (SEM). It is a technique for revealing three-dimensional details on polysaccharide surfaces. Previous research has suggested that extraction, purification, and preparation conditions can affect polysaccharide structures, activity, and surface shape [55]. The surface morphology of CICP’s was evaluated by SEM at magnifications of 1000 and 3000 in Figure 3B. CICP had a smooth surface and no discernible network. CICP’s surface was like hot-water-extracted noni polysaccharides [56].

### 3.6. Chain Conformational Analysis

Congo red could form unique compounds with polysaccharides, had a well-organized three-dimensional structure in solution, was generally triple-helical, and in a spectral scan, the maximum absorption wavelength (λ_max_) would vary [57]. Hydrophobic contacts or strong hydrogen bonds among the polysaccharide and the dye molecule strengthened the complex [58]. Figure 3C depicts the interactions of CICP with Congo red. CICP had a triple-helical construction that was destroyed in an extreme concentration of NaOH solution, as the λmax increased to a maximum when the NaOH concentration was first increased, then dramatically decreased when the NaOH concentration was reduced to 0.2 mol/L, signifying that CICP had a triple-helical confirmation that was damaged in an elevated concentration of NaOH solution [59].

### 3.7. In Vitro Antioxidant Activities of CICP

#### 3.7.1. Free Radical Scavenging Assays

Since no single analysis is sufficient to determine the antioxidant status of CICP, it is more prudent to employ several assays that target various mechanisms. As a result, CICP was tested for antioxidant potential using eleven different methods in the current study. CICP’s antioxidant activity was determined employing radical scavenging, reducing ability methods, chelation ability, and lipid peroxidation methods. CICP’s radical scavenging abilities were tested using ABTS, DMPD, and superoxide radical scavenging assays. The reducing potentials were investigated using three separate in vitro assays: the CUPRAC, FRAP, and phenanthroline methods. Lipid peroxidation inhibition and β-carotene decolorizing assay were used to assess anti-lipid peroxidation.

##### ABTS Cation Radical Scavenging Activity

**Figure 4:**
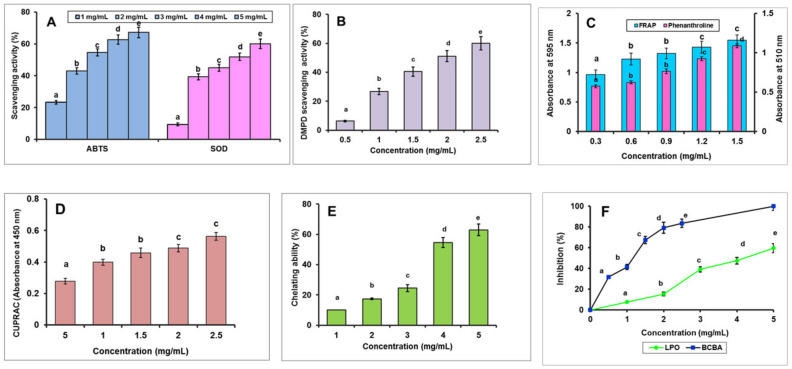
In vitro antioxidant assays of CICP. ABTS and superoxide radical scavenging activity (**A**); DMPD activity (**B**); FRAP and phenanthroline activity (**C**); CUPRAC activity (**D**); chelating ability (**E**); LPO and BCB inhibitory activity (**F**). Results were expressed as mean ± SD (*n* = 3). Mean values within each graph with different letters (a–e) show significant differences (*p* < 0.05) between concentrations.

ABTS+ cation technique is commonly used to assess the antioxidant capability of natural products in vitro. ABTS combines with potassium persulfate to produce the blue-green cation ABTS free radical, and the antioxidant component reacts with the ABTS free radical to bleach the system, indicating CICP’s antioxidant ability. Figure 4A shows the scavenging effect of CICP on the ABTS radical as compared to Trolox. In the concentration range of 1–5 mg/mL, CICP’s scavenging action against the ABTS radical was related to its concentration, with a maximum at 5.0 mg/mL (67.14%). However, CICP’s (EC_50_ = 2.99 mg/mL) ability to scavenge ABTS radical is inferior to that of Trolox (EC_50_ = 0.193 mg/mL) (Table 2). Wang et al. [60] found that the ABTS radical quenching rate of crude *Pleurotus ferulae* polysaccharide and 0.3 M NaCl eluted fraction were 59.63% and 48.16%, respectively, at 3.0 mg/mL, whereas the quenching rate of CICP was comparable to that PFP at the same concentration.

##### Superoxide Radical Scavenging Activity

The superoxide anion is a critical predecessor towards the creation of extremely responsive radicals, for instance, singlet oxygen, hydrogen peroxide, and hydroxyl radicals, that are significant in the oxidation of lipids, proteins, and DNA, as well as causing pathological events. Figure 4A depicts the effects of the superoxide radical scavenging activities of CICP and Trolox. In the concentration range tested (1–5 mg/dL), there was a positive association between CICP concentration and its superoxide radical scavenging behaviour. The superoxide radical trapping efficiency of CICP at 1 and 5 mg/mL was 9.37% and 60.00%, respectively, implying that many superoxide anion radicals were scavenged. CICP has a weaker superoxide radical scavenging activity (EC_50_ = 7.14 mg/mL) than vitamin C (EC_50_ = 0.77 mg/mL) (Table 2). The ability of *Lepista nuda* polysaccharides (LNP) to scavenge superoxide anion free radicals improved as concentration was raised, compared to polysaccharides from other mushrooms. The scavenging activity of crude CICP on superoxide anion radicals was 51.87% higher than that of crude LNP (36.64%), LNP1 (24.67%), and LNP-2 (23.42% at a concentration of 4.0 mg/mL [43]. Crude ORP (35.6%), ORP-1 (13.4%), ORP-2 (9.4%), and ORP-3 (14.4%) all had lesser superoxide scavenging activity [5] than CICP (51.87%) at 4.0 mg/mL. Pursuant to the findings, the method of scavenging superoxide radicals may be linked to the dissociation energy of the OH bond. At the same time, the existence of electrophilic groups such as aldehyde or keto facilitated hydrogen discharge from the OH bond, efficiently scavenging superoxide anions [61].

##### DMPD Radical Scavenging Activity

With a decrease in absorbance, antioxidant compounds that are H-atom contributors to DMPD.^+^ quench the colour of the DMPD.+ solution. CICP’s scavenging activities against DMPD.^+^ radicals improved considerably as concentrations increased (0.5 to 2.5 mg/mL), as shown in Figure 4B. The scavenging efficiency of CICP on DMPD.^+^ radicals was 60.01% at 2.5 mg/mL, which was lower than Trolox (83.48% at 0.2 mg/mL). CICP’s EC_50_ value was 1.99 mg/mL, higher than Trolox (0.008 mg/mL) (Table 2) suggesting that it has moderate DMPD radical scavenging activity. The EC_50_ values of water-soluble polysaccharides from *Dalbergiasissoo Roxb*. (DSLP), *Mimosa diplotricha* var. *diplotricha Sauvalle* (MDSP), and *Tectona grandis* L. (TGBP) were 3.360, 4.980, and 2.360 mg/mL, respectively [61], which were lower than CICP.

#### 3.7.2. Metal Chelation Assay—Ferrous Ions

Metal ions, vital for human well-being, also possess the ability to be poisonous. Fe is the most attractive pro-oxidant among the transition metals because of its elevated reactivity in stimulating and accelerating lipid peroxidation by decaying hydroperoxides to reactive free radicals through the Fenton reaction. Metal chelating behaviour is one of the predominant antioxidant mechanisms because it can absorb catalysing transition metals in biological systems and block radical-mediated oxidative chain events [62]. The capacity of CICP to chelate ferrous ions was calculated in this study by detecting the decrease in absorbance at 562 nm caused by the formation of the ferrozine–Fe^2+^ complex. The chelating strength of CICP improved as its concentration increased, as shown in Figure 4A. The metal chelating activity of CICP increased dramatically at various concentrations (1 to 5 mg/mL), peaking at 62.92% at 5 mg/mL, with an EC_50_ value of 4.11 mg/mL, which is lower than EDTA (EC_50_ = 0.024 mg/mL) (Table 2). When compared to Huangshan floral mushroom (42.68%) [63], Jinqian mushroom (14.06%) [64], *Oudemansiella radicata* mushroom, ORP-2 (37.50%) [5], and *Lepista nuda*, LNP-1 (50.62%) [43], the extracted CICP’s chelating capacity at 5 mg/mL was effective. It has been documented that the existence of functional groups such as -SH, C=O, -NR_2_, -PO_3_H_2_, and -S- is typically correlated with the ability of a compound to chelate ferrous ions [65].

#### 3.7.3. Reducing Potential Antioxidant Assays

##### Cupric Reducing Antioxidant Capacity (CUPRAC)

The CUPRAC approach was used to assess CICP’s ability to reduce Cu^2+^-Cu^+^ by an antioxidant substance. The reducing capacity of CICP steadily improved as the concentration increased from 0.5 to 2.5 mg/mL (Figure 4B). At a dose of 5 mg/mL, the reducing capacity of CICP reached its limit, (Absorbance, 0.563, EC_50_ value = 1.97 mg/mL) but it was still lower than Trolox (EC_50_ = 0.033 mg/mL) (Table 2). The reducing capacity of purified *P. eous* polysaccharide fraction (P3a) improved as the concentration increased (1 to 5 mg/mL), reaching a maximum of 0.991 at 5 mg/mL. The findings on the cupric reducing power of CICP studied suggested that it may have a role in the antioxidant activity noticed.

##### Fe^3+^-TPTZ Reducing Capacity

The capacity of the sample to sustain the transformation of Fe^3+^ to ferrous ions in the existence of TPTZ was determined using the FRAP assay. As the concentration rose from 0.3–1.5 mg/mL, the FRAP absorbance increased steadily (Figure 4C). At a concentration of 1.5 mg/mL, CICP had a FRAP absorbance of 1.545, which was lower than Trolox (0.938 at 0.1 mg/mL). CICP had a higher EC_50_ (2.78 mg/mL) than Trolox (0.04 mg/mL) (Table 2). Two polysaccharide fractions from crude polysaccharides of *Helvella leucopus* (HLP1-1 and HLP2-1) [10] demonstrated weak reducing capacity and were far inferior to CICP.

##### Phenanthroline Assay

The phenanthroline approach is focused on antioxidants’ capability to reduce Fe(III) to Fe(II), which reacted with *o*-phenanthroline to form a red-orange complex. In Figure 4C, the reducing power of CICP and BHT are shown. The reducing capacities increased as the sample concentration increased. CICP had reducing capacities of 0.574–1.088 at 0.3–1.5 mg/mL, respectively. The EC_50_ values for CICP and BHT were 0.78 and 0.15 mg/mL, respectively (Table 2).

#### 3.7.4. Lipid Peroxidation Assays

##### Lipid Peroxidation Inhibition

Lipid peroxidation is the oxidation of polyunsaturated fatty acids in cell membranes, which results in a variety of degradation products. MDA is a lipid peroxidation product that can react with TBA to produce a pinkish red chromogen with a maximum absorption wavelength of 532 nm. The TBARS assay, which uses egg-yolk homogenate as a lipid source, is commonly used to determine antioxidant activity against lipid peroxidation, and the results are shown in Table 2. With an increase in concentration, the LPO inhibition of CICP grew significantly from 1–5 mg/mL (Figure 4D). In comparison to Trolox (EC_50_ = 0.058 mg/mL), the inhibition of CICP is 59.61% at 5 mg/mL, with an EC_50_ of 4.18 mg/mL (Table 2). The LPO inhibition of *Tylopilus ballouii* mushroom hetero polysaccharide is approximately 28%, which is lower than CICP in this research [66].

##### β-Carotene Bleaching Inhibition

The β-carotene bleaching method is based on addition reaction of lipid radical on the C=C double bond of β-carotene, and CICP’s ability to preserve the orange colour of β-carotene is determined by its antioxidant power [67]. With doses ranging from 0.5 to 2.5 mg/mL, the β-carotene bleaching inhibition of CICP ranged from 31.81% to 83.42% (Figure 4D). CICP and Trolox had EC_50_ of 0.560 mg/mL and 0.139 mg/mL, respectively (Table 2). CICP’s EC_50_ value was higher than that of *Armillaria mellea* (EC_50_ = 0.87 mg/mL), *Calocybe gambosa* (EC_50_ = 8.17 mg/mL), and *Coprinus comatus* mushroom polysaccharides (EC_50_ = 7.43 mg/mL) [68]. The high antioxidant activity of CICP indicates that they may be effective in preventing oxidative membrane lipid degradation.

Overall, CCIP’s reducing abilities were found to be more potent with lower IC_50_ values, when compared to other antioxidant assay’s such as radical scavenging, metal chelating, and lipid peroxidation inhibition. Our results are consistent with prior research on the antioxidant activity of acidic polysaccharides from *Craterellus cornucopioides* [69].

### 3.8. In Vitro Anticoagulant Activity

**Figure 5:**
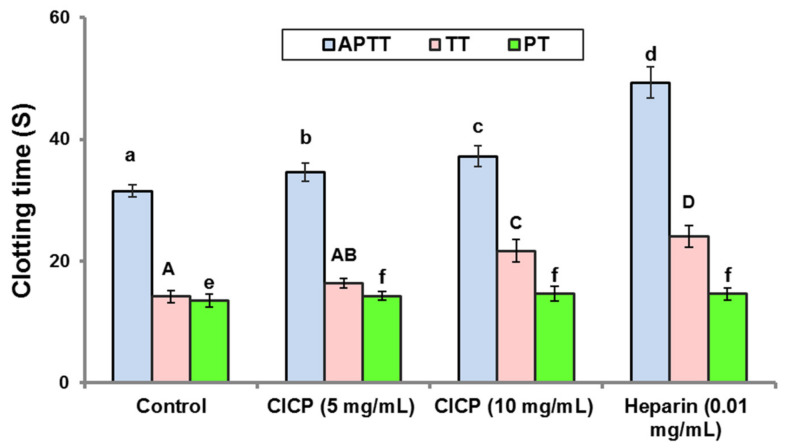
Anticoagulant activity of CICP. Results were expressed as mean ± SD (*n* = 3). Mean values with different letters in each assay (APTT, a–d), (TT, A-D) and PT (e,f) show significant differences (*p* < 0.05) between different groups.

CICP’s in vitro anticoagulant activities were assessed using APTT, TT, and PT, with heparin serving as a reference and control (0.9% saline). APTT is connected with the intrinsic coagulation pathway of plasma, PT with the extrinsic pathway, and TT with the third coagulation pathway. Since the above tests track clot formation, they are regarded as functional tests [70]. In the tested concentration range (5 and 10 mg/mL), no clotting inhibition was found in the PT test of CICP, implying that CICP cannot inhibit the extrinsic pathway of coagulation. In a concentration-dependent manner, CICP may prolong APTT and TT (Figure 5). CICP could inhibit the intrinsic coagulation pathway and thrombin-mediated fibrin formation, based on the prolongation of APTT and TT in comparison to control. The anticoagulant properties of polysaccharides from *Pleurotus eous* [38], *Catathelasma ventricosum mycelia* [71], and *Russula virescens* [72] are consistent with our findings.

### 3.9. Anti-Tumour Activity

**Figure 6:**
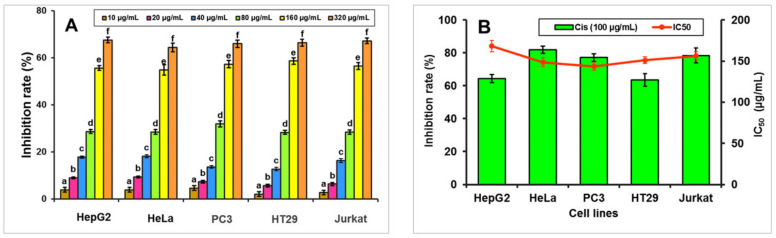
Anti-tumour activity of CICP on different human cell lines (**A**); IC_50_ values (**B**). Mean values within each cell line with different letters (a–f) show significant differences (*p* < 0.05) between concentrations.

MTT assay was utilized to assess the antiproliferative activity of CICP on selected human cells. The inhibition rates of HeLa, PC3, HT29, HepG2, and Jurkat cells increased with concentrations of CICP ranging from 10 to 320 μg/mL, reaching maximum values of 64.32%, 65.93%, 66.39%, 67.51%, and 67.17% respectively at 320 μg/mL. At 100 μg/mL, the positive control (cisplatin) inhibited cells by 81.77%, 77.07%, 63.53%, 64.37%, and 78.42% (Figure 6A). The EC_50_ (μg/mL) values of CICP on different cell lines increased in the order of PC3 (143.60) > HeLa (148.40) > HT29 (151.00) > Jurkat cells (156.30) > HepG2 (168.30) (Figure 6B). Two polysaccharide fractions from *Helvella leucopus*, HLP1-1, and HLP2-1, inhibited HepG2 cells by 26.60% and 62.19%, respectively, at a concentration of 2000 μg/mL [10], which is comparatively lower than CICP. In vitro inhibition of HeLa cell growth by two fractions from *Sarcodona spratus* (PSAN-neutral) and (PSAA-acidic) had EC_50_ values of 289.9 μg/mL and 183.8 μg/mL [73], respectively, which were lower than PepA-1a inhibition on HeLa cells. *Pleurotus djamorvar. roseus* biosynthesized silver nanoparticles inhibited PC3 cell proliferation with an EC_50_ of 10 μg/mL. Polysaccharides anti-tumour efficacy is determined by monosaccharide component, protein content, and molecular mass [74]. Polysaccharides with higher molecular and water solubility have stronger anticancer activity, and carboxylate groups can augment polysaccharide anticancer activity [75]. CICP having carboxylate groups inhibits the growth of various cell lines.

The antioxidant activity of CICP varies with concentration, and it has a scavenging impact on free radicals, reducing power, metal chelating capacity, and lipid peroxidation inhibition. ROS are produced instinctively by metabolism in healthy cells and are counterbalanced by natural antioxidative protection systems. Excess ROS will accumulate and cause oxidative stress if the balance is disrupted. Uncontrolled ROS development can harm DNA, proteins, and lipids, leading to a variety of chronic health issues. As a result, antioxidants that scavenge ROS are beneficial for protecting macromolecules from harm [76].

Polysaccharide bioactivities are strongly linked to structural factors such as monosaccharide content, molecular weight, glycosidic linkage, degree of branching, and chain conformation. Furthermore, the molecular weight of a substance has an impact on its antioxidant activity [77], as well as being influenced by the structure of functional groups (sulphate, uronic acid, etc.). The presence of uronic acid appears to be critical in determining the antioxidant capacity of polysaccharides [78]; the larger the increase in the uronic acid level, the better will be the antioxidant capability [79]. Polysaccharide uronic acid groups can bind to H-atoms on the anomeric carbon atoms. The sulphate group acts as an electrophile, promoting intramolecular hydrogen abstraction and improving polysaccharide antioxidant activity [80]. Previous research suggested that the glucose, galactose, and mannose were significantly related to anticancer activity [81], which is in accordance with the present findings. CICP possessed triple-helix conformation, which is a prime characteristic for anticancer action, as reported by previous authors [82]. Sun et al. [83] studied polysaccharides with varying molecular weights and discovered that those with a low molecular weight had superior anticancer properties. Low molecular weight polysaccharides with more fragmented chains may have improved water solubility and biological activity [84]. Low-molecular-weight polysaccharides are expected to have more deduced OH groups, which can receive and quench free radicals. The α- and β-glycoside linkages were both correlated with anticancer effects [85]. CICP contains α- and β- configurations, which could have improved tumour growth inhibition. Our findings are also consistent with a prior study, that polysaccharides in the pyranose form with a beta structure exhibited increased antioxidant properties [86]. The findings revealed that CICP had the moderate to highest antioxidant activity, which may be due to its low molecular weight (9.371 × 10^4^ Da), high uronic acid content (29.67%), sulphate (4.70%), galactose content (76.40%), and triple helix shape. The relationship between polysaccharide structure and biological function can be complicated. More research into the relationship between polysaccharide structure and function is needed.

## 4. Conclusions

The crude polysaccharide was extracted from *C. indica* using hot water. CICP had little protein and had α- and β- type glycosidic linkages, according to UV and FT-IR spectra. CICP contained galactose as a major monosaccharide and its molecular weight was 9.371 × 10^4^ Da and 2.457 × 10^3^ Da. The FT-IR spectra of CICP revealed polysaccharide-like absorption peaks. Furthermore, CICP has triple-helical conformation. The antioxidant properties of CICP were assessed using a variety of in vitro tests. CICP exhibits significant antioxidant activity, as evidenced by ABTS, DMPD, superoxide radical, ferric reducing power ability, metal chelating capacity, and lipid peroxidation inhibition assays, all of which exhibited a dose-dependent relationship. CICP also inhibited the growth of HeLa, PC3, HT29, HepG2, and Jurkat cells in a concentration-dependent manner. CICP demonstrated moderate APTT and TT prolonging action in anticoagulant activity testing. The molecular weight, monosaccharide composition, sulphate, and uronic acid concentrations, as well as the triple helical shapes of polysaccharides, all contributed to CICP’s biological activity. This investigation lays the groundwork for subsequent research into the relationship between CICP’s structure and activity, as well as polysaccharide development as a natural biological agent for the pharmaceutical and functional food industries.

## Figures and Tables

**Table 1:** Preliminary characteristics of CICP #.

Physicochemical Properties	CICP
Neutral sugar (%)	79.29 ± 5.02
Uronic acid (%)	4.40 ± 0.35
Sulphate (%)	4.70 ± 0.36
Protein (%)	2.15 ± 0.18
Molecular weight distribution (Da)	
Peak 1	9.371 × 10^4^
Peak 2	2.457 × 10^3^
Monosaccharide composition (molar %)	
Xylose	3.06
Arabinose	3.58
Fucose	5.55
Rhamnose	1.15
Mannose	2.93
Galactose	66.23
Glucose	15.52
Glucuronic acid	1.98

# Each value is expressed as mean ± SD obtained from triplicate measurements except for molecular weight and monosaccharide composition.

**Table 2:** EC_50_ (mg/mL) values of CICP and standard compounds in different antioxidant assays.

Antioxidant Assays	CICP	Trolox	Vc	EDTA	BHT
**Radical scavenging activity assay**					
ABTS radical scavenging activity	2.99	0.19	-	-	-
Superoxide radical scavenging activity	3.82	-	0.77	-	-
DMPD radical scavenging activity	1.99	0.03	-	-	-
**Metal chelating assay**					
Ferrous ion chelating activity	4.11	-	-	0.03	-
**Reducing ability assay**					
Potassium ferricyanide ability	2.11	0.78	-	-	-
Cupric ion reducing power ability	1.97	0.03	-	-	-
Fe^3+^TPTZ reducing ability	2.78	0.04	-	-	-
Phenanthroline assay	0.78	-	-	-	0.15
**Anti-lipid peroxidation assay**					
Lipid peroxidation inhibition	4.18	0.06	-	-	-
β-carotene bleaching inhibition	0.56	0.14	-	-	-

**Figure A1:**
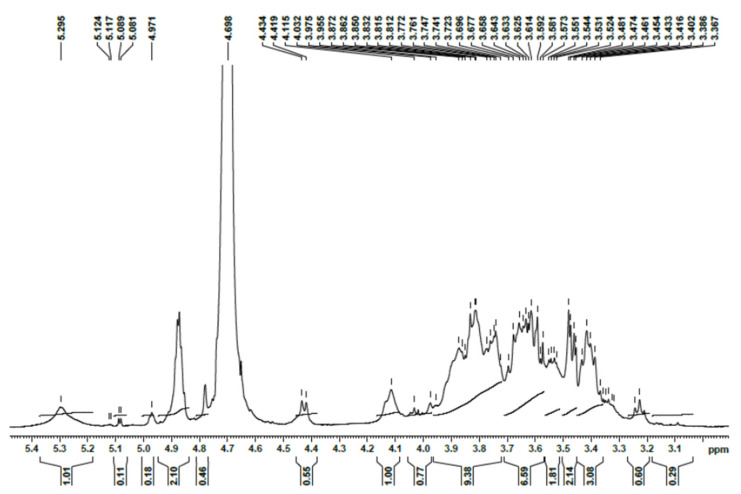
^1^H spectra of CICP.

**Figure A2:**
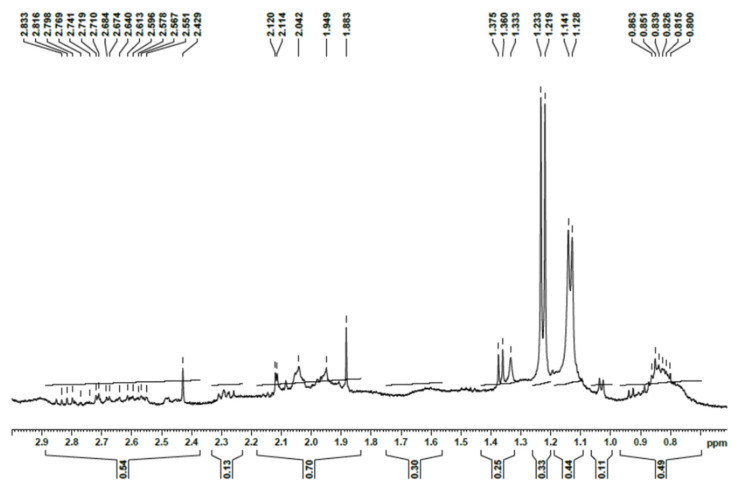
^1^H spectra of CICP.

## Data Availability

Data is contained within the article.
